# A Universal Positioning System for Coupling Characterization of SEM and AFM

**DOI:** 10.1155/2021/5550311

**Published:** 2021-08-12

**Authors:** Jinchao Liu, Andi Wang, Ji Yang, Shiheng Yin, Xianfeng Yang

**Affiliations:** ^1^Analytical and Testing Center, South China University of Technology, Guangzhou 510641, China; ^2^School of Chemistry and Chemical Engineering, South China University of Technology, Guangzhou 510641, China

## Abstract

Hyphenated techniques, providing comprehensive information in various aspects such as constituent, structure, functional group, and morphology, play an important role in scientific research. Nowadays, coupling characterization of the same position in microscale is in great need in the field of nanomaterial research and exploration. In this article, a new hyphenated technique was developed to facilitate the coupling characterization of atomic force microscope (AFM) and scanning electron microscope (SEM) by designing a universal positioning system. The system consisted of a specimen holder with coordinate grids and a software for converting the coordinate values of the same point to fit SEM, specimen holder, and AFM system. In working condition, the coordinates of the labeled points and target position were firstly extracted from the SEM operation software, then converted into the numerical values adapted to the specimen holder itself, and finally transformed into the coordinates matching the AFM system. The experimental result showed that a retrieving rate of 96% was achieved for a spherical target with a diameter of 1 *μ*m in a 30 *μ*m × 30 *μ*m square. The hyphenated technique is a universal, accurate, efficient, and financially feasible method in microanalysis field and has great application potential.

## 1. Introduction

Hyphenated techniques have been widely used in the field of active ingredient analysis and chemical reaction mechanism revelation. Owning to the benefits of coupling characterization of gas chromatography-mass spectrum (GC-MS), liquid chromatography-mass spectrum (LC-MS), liquid chromatography-Fourier transform infrared spectroscopy (LC-FTIR), and so on, researchers can obtain a great deal of useful information about composition, structure, and/or functional groups of the samples to accelerate their research work [1, 2].

In the last decade, a hyphenated technique in the field of microanalysis became a new research hotspot. Visual and quantitative coupling characterization results in micro- or submicroscale provided crucial information to discover and understand the operation rules in the microscopic world [3]. Ando et al. summarized the recent hyphenated technique of optical microscopy, electron microscopy, atomic force microscopy, and fluorescence microscope in the field of biomedical, chemistry, and catalysis and presented many correlative imaging results from 2D to 3D [4]. Among all of the instruments, AFM and SEM are two essential instruments applied in microanalysis. AFM was invented in 1986 for the purpose of obtaining three-dimensional features of the sample surface in nanoscale [5]. Nowadays, the functions of AFM have been extended to quantitative nanomechanical analysis, characterization of electric/magnetic field, surface potential, and scanning electrochemical measurement (Fig. S1) [6–8]. Above all, the open and diverse working environment (e.g., vacuum, atmospheres, or solutions) enables AFM to serve as an ideal platform for in situ characterization. Up to date, many outstanding works have been concentrated upon the AFM-related coupling characterization. Boerio and Starr [7] reported a new technique which combined the characterization of AFM and FTIR to give the morphology and functional group changes of polystyrene and polycarbonate films after the infrared absorption. The temperature change of the polymer surface was also tested by the scanning thermal microscopy probe of AFM spontaneously. Gayathri [8] reviewed the application of coupling characterization of AFM-thin-film attenuated total reflectance/ultraviolet resonance Raman spectroscopy in the kinetics of formation and structural characterization of tau fibrils.

Although widely employed in nanoscale characterization, AFM encounters obstacles in applications [9]. It is usually difficult to search targets in AFM's optical visual field when the targets are micro- or submicroscale due to the uneven distribution of samples, poor contrast between samples and substrate, discontinuous optical visual field trace, and limited scanning area. Taking a Bruker Icon AFM for instance, the visual field of the optical microscope is 540 *μ*m × 520 *μ*m, while the maximum scan range is 100 *μ*m in *X*/*Y* direction and 13.4 *μ*m in *Z* direction [10]. Consequently, in practice, the testing fields are randomly selected, and to some extent, the results are not always representative. Up to now, it is still unable to completely eradicate the phenomenon of taking a part for the whole.

SEM is another requisite in microanalysis. The microtopography, as well as the distribution of surface elements and grain orientation, can be acquired by the SEM equipped with Energy Dispersion Spectrum (EDS) detector and Electron Back-Scattered Diffraction (EBSD) detector (Fig. S2) [11, 12]. In addition, one can obtain a magnified image from micrometer scale to nanometer scale continuously by SEM, which can help researchers to recognize the overall information of the specimen and trace the specific structure at the same time. Nowadays, a high-resolution image of insulating specimen with original surface can be obtained easily by SEM with low accelerate voltage operation mode. After the SEM test, the original state of the specimen can be reserved to conduct the successive characterizations. Nevertheless, the SEM must work in vacuum or low-pressure conditions. Therefore, samples containing volatile are forbidden in SEM. Besides, complex electric and magnetic field in the narrow chamber makes it difficult to execute in situ experiment in SEM.

To combine the advantages of AFM and SEM, various coupling characterization technologies of AFM and SEM have been developed in recent years [12–15]. These technologies can be divided into two categories. One is called in situ test—an AFM is installed in the SEM chamber. This kind of setup can test the same microcosmic area directly in the same environment. Kreith et al. [13] reported a versatile AFM integrated with a SEM and its application in the research of slip steps emanating around nanoindents in single crystalline brass. The work gave the plastic deformation occurring during the in situ nanoindentation for the first time. In 2018, the NenoVision released a commercial AFM stage—LiteScope™ in which the AFM part can match various types of SEMs with a communication cable through a tunnel [14]. The product facilitated the application of coupling characterization of AFM and SEM.

However, the shortcomings of the in situ technique are also prominent. First, AFM operation in the vacuum bin is very inconvenient; second, resolution reduction is inevitable due to the overlarge working distance in the SEM resulted from the existence of AFM probe; last, the complex electromagnetic environment in the vacuum chamber may affect the test result of AFM. The high cost of the setup also hinders the application of this technique.

The other category is to test the same position of the specimen in AFM and SEM separately. Loïc et al. [15] reported the work of metrological characterization of a sphere in three dimensions through a combined result of AFM and SEM. The author fabricated specific symbols on the specimen holder to help to recall the same position when the sample was transferred to another instrument. However, the repositioning of the same set of nanoparticles in their paper was delivered from the image comparison and object identification, which is time consuming. Li et al. [16] reported the nanoscale pore structure and mechanical properties of coal through a coupling characterization of AFM and SEM. The sample was tested in AFM firstly and then transferred to SEM. In order to ensure the accurate correspondence between AFM and SEM, the author scanned a huge amount of SEM images and mosaicked them into a much larger imaging range than that obtained in AFM. The whole procedure is inefficient, which would limit its practical applications.

Therefore, it is urgent to develop a compatible, financially feasible, and efficient system to promote the utility of coupling characterization of SEM-AFM. In this article, a new hyphenated technique for the coupling characterization of SEM-AFM was developed. The positioning algorithm applied in the technique takes the points or vectors on the specimen holder as reference to mark the target position. So, the excellent compatibility of the new hyphenated technique to most of the commercial SEMs and AFMs can be achieved. The coordinates of the target position in a new system can be calculated directly through the homebrew software (or Microsoft Excel), which makes the target retrieving process an efficient and accurate one.

Polished silicon wafers, cleavage micas, and copper mesh are all suitable materials for specimen holders which can be printed with coordinate grid by photolithography technique [17–19]. Polished silicon wafers printed with specific patterns were employed in this article. The average cost of each specimen holder is comparable to Cu grids adopted in TEM experiment, making it affordable for most researchers. The coupling characterization results of SiC and Bi_9_O_7.5_S_6_ nanomaterials have achieved the expected results. The comprehensive information obtained from the same position in SEM and AFM provided critical clues in determining the growth mechanism of the SiC nanobelts [20] and photocatalytic properties of the Bi_9_O_7.5_S_6_ nanoflake [21], respectively.

## 2. Materials and Method

### 2.1. Reposition System Setup

#### 2.1.1. Pattern Design on the Specimen Holder

In order to retrieve the target point, we need to define a series of labeled points on the specimen holder. The labeled points should be noncollinear and prominent in both SEM and optical microscopy (affiliated to AFM) visual fields. The correlation between the labeled points and target point helps to position the target point.

Specifically, a polished and doped polycrystalline silicon wafer with a home-designed pattern consisting of molybdenum grids was employed as a specimen holder. Specifications and parameters of the silicon wafers are listed in Table S1. Molybdenum grids with a thickness of 100 nm were manufactured by magnetron sputtering.

**Figure 1:**
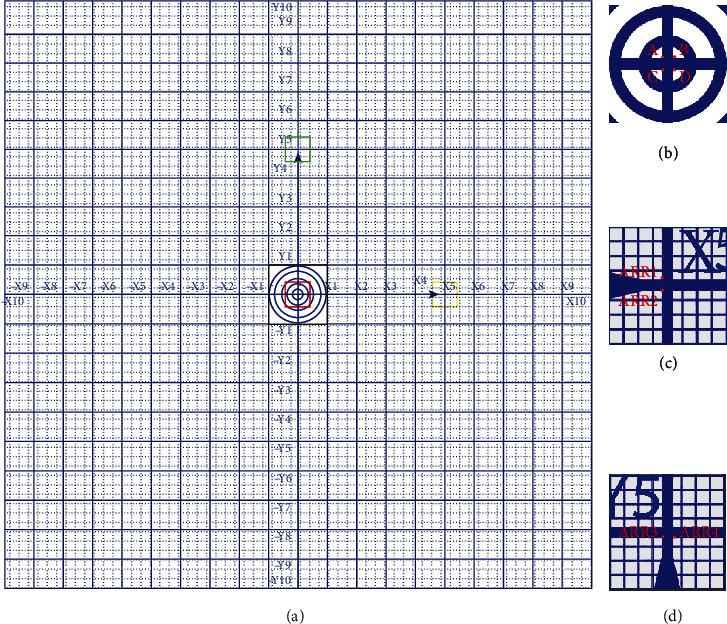
(a) Schematic illustration of the pattern on a specimen holder, the side length of the square is 10 mm, and the thickness of the silicon wafer is 2 mm. (b), (c), and (d) are the magnified images in the boxes of red, yellow, and green, respectively. To be noticed, only the blue lines and symbols in (a) are printed on the specimen holder. All the red labels in (b–d) (e.g., A, B, C, and D on the intersection of *X*- and *Y* -axis line, ARR1~2 and ARR3~4 at the opposite sides of *X*- and *Y*-axis lines near the arrow tips) are only shown in this illustration for expression in convenience, rather than printed on the holder.

The silicon wafers were cut into squares of 10 mm × 10 mm and 2 mm in thickness. Although many patterns can be applied [22–24], our pattern was designed to be a rectangular coordinate system to adapt SEM and AFM system. The pattern on the specimen holder is illustrated as shown in Figure 1. Two perpendicular lines with the width of 50 *μ*m travel through the center of the silicon wafer, which are defined as *X*- and *Y*-axis, respectively. The arrow placed on *X*/*Y*-axis indicates the positive direction. The silicon square is divided into four parts by *X*/*Y*-axis, and each part is equally divided into 100 pieces of smaller squares by the separator lines. The intersections of the separator lines and *X*/*Y*-axis are labeled with ±*X*1 ~ *X*10 and ±*Y*1 ~ *Y*10 sequentially.

#### 2.1.2. Construction of the Coordinate System on Specimen Holder

*(1) Definition of Origin and Unit Vector in *X*/*Y* directions on the Specimen Holder*. In order to obtain an accurate numerical value of coordinate, the origin *O* is defined as the center of the intersection of *X*- and *Y*-axis line rather than printed on the specimen holder. It can be easily calculated from the coordinates of A, B, C, and D labeled on the vertexes of the square intersection of *X*- and *Y*-axis line as shown in Figure 1(b). Likewise, the midpoints of line segments ARR1-ARR2 and ARR3-ARR4 are marked as *H* and *V*, respectively. Herein, the points of ARR1~2 and ARR3~4 are labeled on the opposite sides of *X*- and *Y*-axis near the arrow tips, respectively, as shown in Figures 1(c) and 1(d). The directions of vectors $\overset{⃑}{OH}\;$and $\overset{⃑}{OV}$ are the directions of *X*-axis and *Y*-axis, respectively. The unit vector in *X* direction is$\;\overset{⃑}{OH}/\left|\overset{⃑}{OH}\right|$, in *Y* is $\overset{⃑}{OV}/\left|\overset{⃑}{OV}\right|$.

*(2) Coordinates of Labeled Points and Target Point in the SEM System*. The coordinates of a target point *T*, as well as the labeled points on the specimen holder (A, B, C, and D, ARR1~4) can be read from the stage navigation part of the SEM operation software directly (see in Fig. S3), which are the initial coordinates of our positioning system. The coordinate values of origin *O* are calculated from the average of points A, B, C, and D. Similarly, the coordinate of *H* is calculated from the average of ARR1 and ARR2; the coordinate of *V* is calculated from the average of ARR3 and ARR4. Consequently, the coordinate of $\overset{⃑}{OH}$, $\overset{⃑}{OV}$, $\overset{⃑}{OT}$, $\overset{⃑}{OH}/\left|\overset{⃑}{OH}\right|$, and $\overset{⃑}{OV}/\left|\overset{⃑}{OV}\right|$ can be calculated according to the rule of vector arithmetic.

#### 2.1.3. Correlation between the Target Point and Labeled Points

**Figure 2:**
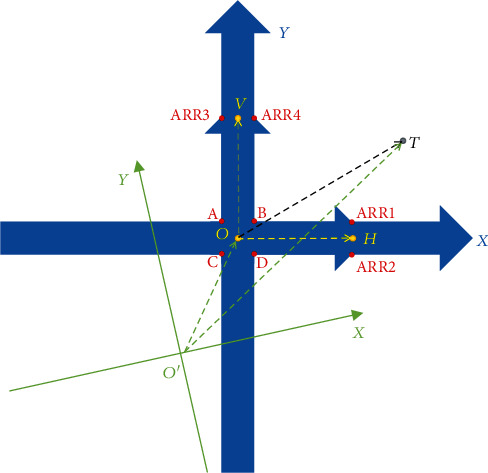
Illustration of the coordinate system on specimen holder and in AFM. The red points are the positions of labeled points; *T* is the position of the target. The point *O* is the center of square ABCD, also defined as the origin of the coordinate system on specimen holder; points *H* and *V* are the midpoint of segment ARR1-ARR2 and ARR3-ARR4, respectively. The direction of vectors $\overset{⃑}{OH},\overset{⃑}{OV}$ is defined as the directions of *X*-axis and *Y*-axis, respectively. *O*′ is defined to be the origin of the coordinate system in AFM (displayed in light green lines to indicate that the positioning system can work even if the directions of the coordinate system of the specimen holder and AFM are not coincident to each other). The vectors of $\overset{⃑}{OH},\overset{⃑}{OV},\overset{⃑}{OT},$$\overset{⃑}{O\prime T},$ and $\overset{⃑}{O^{\prime }O}$ are displayed in dash lines.

Since the specimen holders adopted in the system are flat and clean, the calculation based on the principle of vector addition in a two-dimensional plane is applicable [25]. As shown in Figure 2, there are four coplanar points *O*, *H*, *V*, and *T*. Assuming that the correlation of vectors of $\overset{⃑}{OH}$, $\overset{⃑}{OV}$, and $\overset{⃑}{OT}\;$shown in equation (1) is satisfied in the coordinate system printed on the specimen holder, equation (1) still stands when the numerical value of the vectors is expressed in coordinates of SEM or AFM based on the properties of vector addition [26].

(1) $$\begin{array}{c} \vec{OT}=a\frac{\vec{OH}}{\left|\vec{OH}\right|}+b\frac{\vec{OV}}{\left|\vec{OV}\right|}. \end{array}$$

The coefficients *a* and *b* are unknown numbers which can be worked out according to equation (1) by solving a linear equation in two unknowns.

#### 2.1.4. Retrieve of the Target Point in the AFM System

When transferring to the AFM system, the coordinates of the labeled points on the specimen holder should be transformed into the values adapted to the AFM system.

*(1) Acquisition of the Coordinates of Labeled Points in the AFM System*. The labeled points which can be identified in optical microscopy were placed right under the AFM probe by adjusting the stage and scan range. The repositioning accuracy is low in this step due to the poor resolution of the optical microscopy. In order to improve the positioning accuracy, the label points are scanned in AFM to confirm the accurate position and then the OFFSET function in the AFM operation system is invoked to give accurate numerical position information. The OFFSET function is a function in the AFM operation system, which can move the point to the center of the scan range in a two-dimensional plane through an inverse piezoelectric effect [9]. The distance moved in *X* and *Y* directions are displayed in the software interface (see in Fig. S4(b)). The function can improve the repositioning accuracy to the scale of a nanometer by moving the point through the deformation of piezoelectric ceramic [9].

The final coordinates of the labeled point are the sum of the coordinate value extracted from the AFM stage navigation system (*X* and *Y* values in the red box in Fig. S4(a)) and the corresponding OFFSET value in the AFM operation software interface (*X* and *Y* values in the red box in Fig. S4(b)).

*(2) Solution of Coordinates of $\vec{OT}$ and Point *T* in the AFM System*. Use the coordinates of the labeled points obtained from the foregoing steps to calculate the coordinates of $\overset{⃑}{OH}/\left|\overset{⃑}{OH}\right|$ and $\overset{⃑}{OV}/\left|\overset{⃑}{OV}\right|$ and $\vec{OT}$ according to equation (1).

The origin of the coordinate system in AFM is defined as *O*′ (shown in Figure 2). The coordinate of point *T* in the AFM system is equal to the coordinate value of $\vec{O\prime T}$ which can be calculated according to

(2) $$\begin{array}{c} \vec{O\prime T}=\vec{O\prime O}+\vec{OT}. \end{array}$$

The coordinate of $\vec{O\prime O}$ equals to the numerical values of point *O*, which can be delivered from the AFM system according to the previous section.

Hereto, the coordinate values of the target in the AFM system are worked out.

*(3) Navigation of the Target Point to the Probe*. Navigation can be fulfilled through the stage navigation function in AFM operation software; the steps are shown as follows: (1) input the coordinate of *T* into the stage navigation system and (2) click “Relative Motion” button to move the target point to the position under the probe.

**Figure 3:**
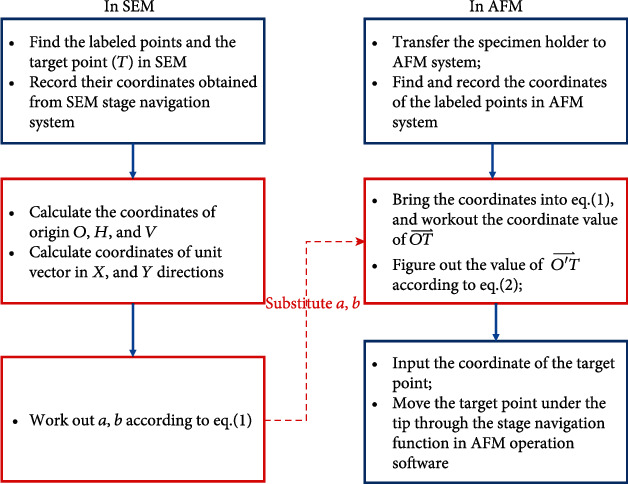
Flow diagram of the reposition process (the steps in blue boxes should be accomplished manually; the steps in red boxes can be finished by the homebrew software explored for the positioning system.)

The whole flow chart is listed in Figure 3.

To obtain the target coordinates more efficiently, we explored a software to calculate the result data according to the above procedure using C language under the frame of Visual Studio 2013. A given set of coordinates of the labeled points and target point in the SEM and AFM system was listed in the homebrew software interface (see in Fig. S5).

### 2.2. Holder and Material Preparation

The whole manufacture progress of specimen holders was assigned to Guangzhou New Vision Company. The lithography machine (SSA600/20, Shanghai Microelectronics Equipment Co. LTD) with a resolution of 100 nm was employed.

The polystyrene (PS) spheres (product No. 6-6-1000) used in repositioning were purchased from Tianjin Baseline ChromTech Research Center. The SiC nanobelt sample was prepared following the procedure illustrated by Chu et al. [20]. The Bi_9_O_7.5_S_6_ nanoflake sample was synthesized following the hydrothermal method described by Meng et al. [21].

### 2.3. Characterizations

A Zeiss Merlin SEM equipped with an Oxford X-MaxN20 energy-dispersive X-ray spectroscopy (EDX) detector and a Bruker Icon AFM was adopted for the repositioning test and coupling characterization of SiC nanobelt and Bi_9_O_7.5_S_6_ nanoflake. For surface potential measurement of Bi_9_O_7.5_S_6_ nanoflake responding to illumination, the KPFM (Kelvin probe force microscopy, amplitude modulate, probe model: SCM-PIT, Bruker) mode was chosen in the AFM operation software, and a mercury lamp (Model: G30T8, Sankyo Denki, Japan) with a wavelength of 254 nm was employed as the UV light resource.

During repositioning procedure, the number of coordinate values in the SEM system was denoted with the unit of a micrometer. The units in the specimen holder and AFM coordinate system were all kept consistent. The numerical values of the labeled points on the specimen holder coordinate system can be calculated from the values from the SEM system as long as the origin point, direction of *X*- and *Y*-axis, and length unit were defined. Only the 2D coordination system is employed in the repositioning by reason of the very limited roughness, and surface bending value changes in the whole test process as shown in Table S1.

## 3. Results and Discussion

### 3.1. Accuracy of Repositioning

To test the reposition accuracy of the system, reposition of a polystyrene (PS) sphere with a nominal diameter of 1 *μ*m was executed. The experiment was executed following the flow diagram listed in Figure 3.

**Figure 4:**
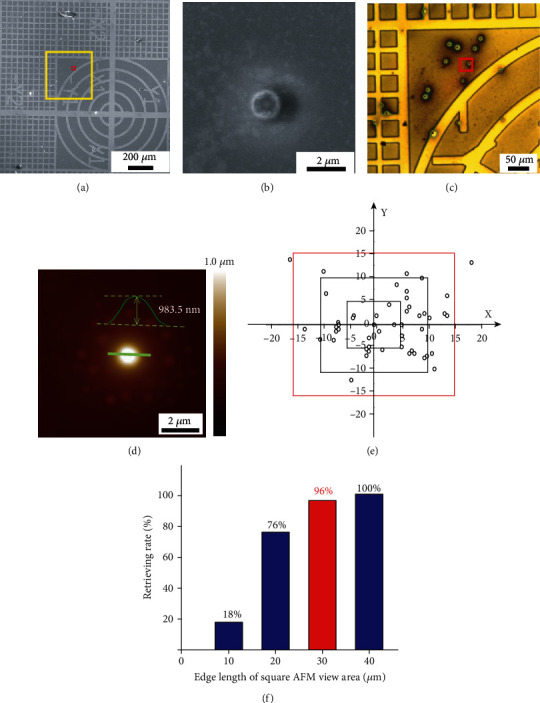
(a) A low magnification SEM image of a part of the specimen holder. (b) SEM image of the target PS sphere, selected from the red square area in (a). (c) Optical image of a part of the specimen holder obtained by the optical microscope affiliated to AFM, corresponding to the yellow square area in (a). (d) An AFM image of the same target sphere as shown in (b). (e) Error distribution in *X* and *Y* directions in the 50 times of the above-selected target retrieving under AFM, unit of the numbers on *X*- and *Y*-axis is *μ*m. (f) The relationship of retrieving rate to edge length of square AFM view area.

First, the related coordinates corresponding to the labeled points as mentioned in Figure 2 were recorded under SEM observation of the specimen holder with PS spheres. Simultaneously, a low magnification SEM image of a typical area was taken as shown in Figure 4(a). Then, we zoomed into a 10 *μ*m × 10 *μ*m square area to select a PS sphere as the target arbitrarily and recorded its corresponding coordinate (Figure 4(b)).

Second, we transferred the holder to AFM to retrieve the target PS sphere according to the procedure mentioned above.  Figure 4(c) is an optical image of the specimen holder corresponding to the yellow square area in Figure 4(a), which is delivered from the optical microscope affiliated to AFM. The target PS sphere was finally found, and a high-resolution AFM image has been taken as shown in Figure 4(d). The height of the sphere was also depicted with green curve in the image, which could not be obtained under SEM directly.

After the first scanning of the target position in AFM, the OFFSET function was executed to evaluate the distance between the target point and the center of the scan area. In the article, the OFFSET value in *X* and *Y* directions was denoted as error. The experiment was repeated 50 times to test the repeatability.  Figure 4(e) shows the error distribution in the range of 30 *μ*m × 30 *μ*m (the red square in Figure 4(e)). In forty-eight out of fifty times, the target can be found in a 30 *μ*m × 30 *μ*m square. In other words, the retrieved possibility for the target point in the scan range of 30 *μ*m × 30 *μ*m is 96% (the highlighted column shown in Figure 4(f) in red).

In order to estimate the repositioning accuracy when the target is far away from the labeled points, we executed the repositioning process 10 times taking a PS sphere in the grid of *X*8‐*Y*8 as the target. The results shown in Table S2 presented that the error distribution near *X*8‐*Y*8 was similar to the occasion that the target next to the center of the grid.

Currently, our repositioning system can hardly achieve the accuracy of a nanometer. A similar report published recently [27] pointed out that stage accuracy, or instrument stability, could be the limiting factor for relocalization accuracy. In our experiment, we also found that the error delivered from the instrument stability was one of the main errors in the repositioning system, which was supported by the stage reproducibility and accuracy test executed in SEM and AFM independently (see in Table S3).

### 3.2. Demonstration of the System

The hyphenated technique has been employed in many experiments. Two typical results were illustrated here.

#### 3.2.1. Measurement of the Step Width and Height in a SiC Nanobelt

Taking the rational synthesis of SiC nanostructures as prototype, Chu et al. [20, 28] proposed a new dislocation growth model to interpret the formation of the nanobelts based on the lowest energy principle in the crystal growth. The information of stepwise spiral terraces in three dimensions is necessary for exploring the growth mechanism. However, the random orientation and intertwined state of the nanobelts on the specimen holder were great challenges in AFM characterization.

**Figure 5:**
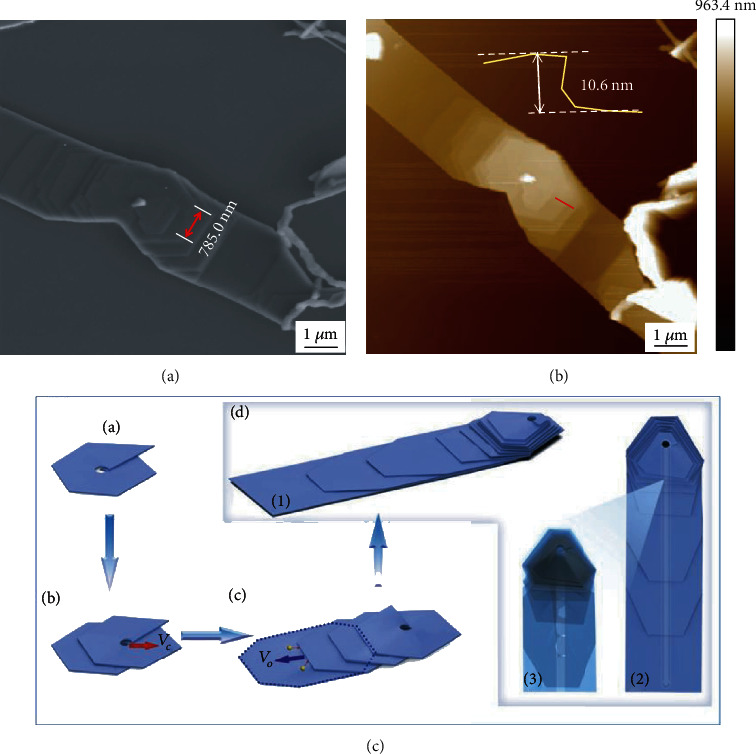
(a) SEM and (b) AFM images of SiC nanobelt at the same position. (c) Schematic illustration of growth mechanism of the nanobelt.

Herein, our system has been employed to solve this problem perfectly. SEM images of the target on a patterned grid with different low magnifications were displayed in Fig. S6. As illustrated in Figure 5, we found a lying down SiC nanobelt as the target and obtained accurate data of terrace width in SEM at first. Then, we retrieved the target in AFM and gained step height data of the same terrace. The combined information provided important supporting evidence for the growth mechanism [20].

#### 3.2.2. Coupling Characterization of a Complex of Bi_9_O_7.5_S_6_

In recent years, Bi-containing compounds have attracted enormous attention due to their unique properties in photocatalysis [21, 29]. The correlation between the elemental composition and photoelectric properties on the same nanostructure needs to be clarified to provide guidance for the controllable synthesis [30, 31]. KPFM technology is an AFM test method base on tapping mode which can give the information of contact potential difference (CPD) [32]. Testing the CPD difference of photocatalysts before and after illumination with the same tip under a specific wavelength is an effective way to evaluate photocatalysts [33]. In our experiment, the surface topography, composition, and the photoelectrical properties of Bi_9_O_7.5_S_6_ nanoflake were characterized through the hyphenated technique executed by SEM and AFM.

**Figure 6:**
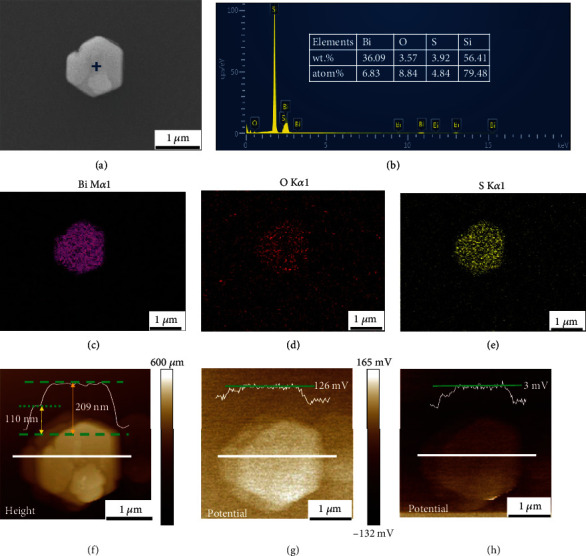
SEM and AFM characterization of the same Bi_9_O_7.5_S_6_ nanoflake: (a) SEM image of the nanoflake. (b) EDS spectrum and elemental composition of the same nanoflake. (c–e) EDS mapping: spatial distribution of Bi, O, and S elements. (f) AFM height image of the same area, the white curve showed the height of the area marked by the white line. (g, h) KPFM test of the same nanoflake: showing surface potential response to illumination of 254 nm ultraviolet light OFF (g) and ON (h), the white curves showed the potential of the area marked by the white line.

The comprehensive information about the nanoflake is shown in Figure 6. The topographic and composition of the nanoflake obtained by SEM helped to speculate the crystal type (see in Figs. S7 and 6(a)–6(e)). The AFM height image (Figure 6(f)) gave the information about the thickness of a single nanoflake (~110 nm). The KPFM test gave the information about the surface potential. Figures 6(g) and 6(h) showed the surface potential changes response to the UV with a wavelength of 254 nm (Figures 6(g) and 6(h)). A drop of 123 mV in surface potential is observed which can be attributed to the charge separation at the surface during the illumination [33, 34]. The coupling characterization provides integrated information in the exploration of the structure-function relationship [33].

## 4. Conclusion

In this work, a universal positioning system that facilitated the hyphenated technique of the coupling characterization of AFM and SEM was presented. Despite the simplicity of the positioning arithmetic, a retrieving rate of 96% with a sphere of 1 *μ*m in a scan size of 30 *μ*m × 30 *μ*m is achieved. The successful instances of SiC nanobelt and Bi_9_O_7.5_S_6_ nanoflakes utilizing coupling characterization of SEM and AFM have demonstrated the compatibility, efficiency, and accuracy of this hyphenated technique. This remarkable performance was enabled by the introduction of stage navigation function of SEM and AFM which improve the accuracy of the coordinates of the labeled and target points. The OFFSET function in the AFM operation system also plays a key role in optimizing the positioning system performance. This is an important progress towards the low-cost, efficient, and accurate application of coupling characterization of SEM and AFM which will speed up the research in microworld in the future.

## Data Availability

No data were used to support this study.

## Supplementary Materials

Supplementary Materials Table S1: parameters of the polished silicon wafer [1]. Table S2: the reposition accuracy for the target in X8-Y8 grid. Table S3: the accuracy and reproducibility of the stage navigation system in SEM and AFM. Note: the average of the data is the average of the absolute value of the data in each column. The standard deviation is the standard deviation of the absolute value of the data in each column. The average of the data in each column of the table reflected the accuracy of the stage navigation system. The standard deviation of the data in each column reflected the reproducibility of the stage navigation system. Fig. S1: schematic diagram of working principle of AFM [2] and the typical results [3]. (A) Working principle of AFM. (B) The capacitance gradient and contact potential difference (CPD) distribution of spines of neurons using EFM and KPFM technologies, respectively: (a) the topography of a spine in a neuron; (b) the corresponding capacitance gradient image of (a) obtained from EFM test; (c) the topography of a spine in a neuron; (d) the corresponding CPD image of (c) obtained from (d). Reproduced with permission from Ref. [3]. For interpretation of the references to colors in this figure legend, the reader is referred to the web version of this article. Fig. S2: schematic diagram of the structure of SEM [4] and the typical results [5]. (A) Working principle of SEM. (B) Typical results obtained from SEM and accessories (BSE, EBSD, and EDS): (b1) phase contrast, (b2) inverse pole figure (parallel to *Y*-axis), and (b3) EDS maps of Al-Mn particle in LT-Al_11_Mn_4_. Reproduced with permission from Ref. [5]. For interpretation of the references to colors in this figure legend, the reader is referred to the web version of this article. Fig. S3: SEM images and the coordinate values of the labeled points (A, B, C, D, ARR1~4) and target point (T) in the SEM system. Fig. S4: interface of the navigation part (a) and scan part (b) of AFM operation software. Fig. S5: interface of the homebrew software explored for the positioning system: a given set of coordinate values of labeled points and target point in SEM (i) and AFM (ii) system was displayed. Fig. S6: SEM images of the object shown in Figure 5 with different magnifications. Fig. S7: SEM images of the object shown in Figure 6 with different magnifications.
