# Supplementary material for: A Universal Positioning System for Coupling Characterization of SEM and AFM
Source: Scanning. 2021 Aug 12;2021:5550311. doi: 10.1155/2021/5550311 (PMC8376456; doi:10.1155/2021/5550311)
Supplement: Supplementary Materials — Table S1: parameters of the polished silicon wafer [1]. Table S2: the reposition accuracy for the target in X8-Y8 grid. Table S3: the accuracy and reproducibility of the stage navigation system in SEM and AFM. Note: the average of the data is the average of the absolute value of the data in each column. The standard deviation is the standard deviation of the absolute value of the data in each column. The average of the data in each column of the table reflected the accuracy of the stage navigation system. The standard deviation of the data in each column reflected the reproducibility of the stage navigation system. Fig. S1: schematic diagram of working principle of AFM [2] and the typical results [3]. (A) Working principle of AFM. (B) The capacitance gradient and contact potential difference (CPD) distribution of spines of neurons using EFM and KPFM technologies, respectively: (a) the topography of a spine in a neuron; (b) the corresponding capacitance gradient image of (a) obtained from EFM test; (c) the topography of a spine in a neuron; (d) the corresponding CPD image of (c) obtained from (d). Reproduced with permission from Ref. [3]. For interpretation of the references to colors in this figure legend, the reader is referred to the web version of this article. Fig. S2: schematic diagram of the structure of SEM [4] and the typical results [5]. (A) Working principle of SEM. (B) Typical results obtained from SEM and accessories (BSE, EBSD, and EDS): (b1) phase contrast, (b2) inverse pole figure (parallel to Y-axis), and (b3) EDS maps of Al-Mn particle in LT-Al11Mn4. Reproduced with permission from Ref. [5]. For interpretation of the references to colors in this figure legend, the reader is referred to the web version of this article. Fig. S3: SEM images and the coordinate values of the labeled points (A, B, C, D, ARR1~4) and target point (T) in the SEM system. Fig. S4: interface of the navigation part (a) and scan part (b) of AFM operation software. Fig. S5: interf [file 5550311.f1.docx]

Support Information

Specification

1. The models of SEM and AFM adopted herein were Zeiss Merlin and Bruker Icon respectively.

2. The lithography machine is SSA600/20（Shanghai microelectronics equipment Co. LTD）with a resolution of 100 nm. The whole manufacture progress of the specimen holders was assigned to Guangzhou New Vision Company.

3. Only 2-D coordination system is employed by reason of the very limited Z value changes in the whole test process.

4. The view field of the optical microscopy in AFM system is 500 um*500 um.

The size of scanning area is set to be 30 um*30 um in AFM which is adequate for searching target point. Comparison of SEM and AFM images can help to identify the target point. Once the target point was found, zoom scanning with higher resolution or more information can be executed.

5. The numbers of coordinate values in the SEM system was denoted with the unit of micrometer. The unit in specimen holder and AFM coordinate system were all kept consistent.

The numerical values of the labeled points on the specimen holder coordinate system can be calculated from the values from SEM system as long as the origin point, direction of X, Y axis, and length unit were defined.

6. The SiC nanobelt sample was prepared follow the procedure illustrated by Chu et al ^[1]^. The Bi_9_O_7.5_S_6_ nanoflake sample was synthesized following the hydrothermal method described by Meng et al ^[2]^.

7. Mercury lamp (Model: G30T8, Sankyo Denki, Japan) was the UV light resource with a wavelength of 254 nm.

Table

Table 1 Parameters of the polished silicon wafer

| Thickness | 525 um |
| --- | --- |
| Diameter | 101.6 $\pm$ 0.1 mm |
| Doping Type | N type |
| Electrical Resistivity | 15 |
| Roughness | < 0.5 nm |
| TIR (Flatness) | < 3 um |
| TTV (War page) | < 10 um |
| Surface Bending | <10 um |

Figures

Fig.S1 Schematic diagram of working principle of AFM and the typical results. (a) Working principle of AFM; (b)~(d) images of nanotubes on a gold surface: height image (b), Kelvin Probe Force Microscopy (KPFM) image (c), Electrical Field Microscopy (EFM) image (d); (e)~(f) images of magnetic tape: height image (e) and Magnetic Field Microscopy (MFM) image (f) ^[3,4]^.


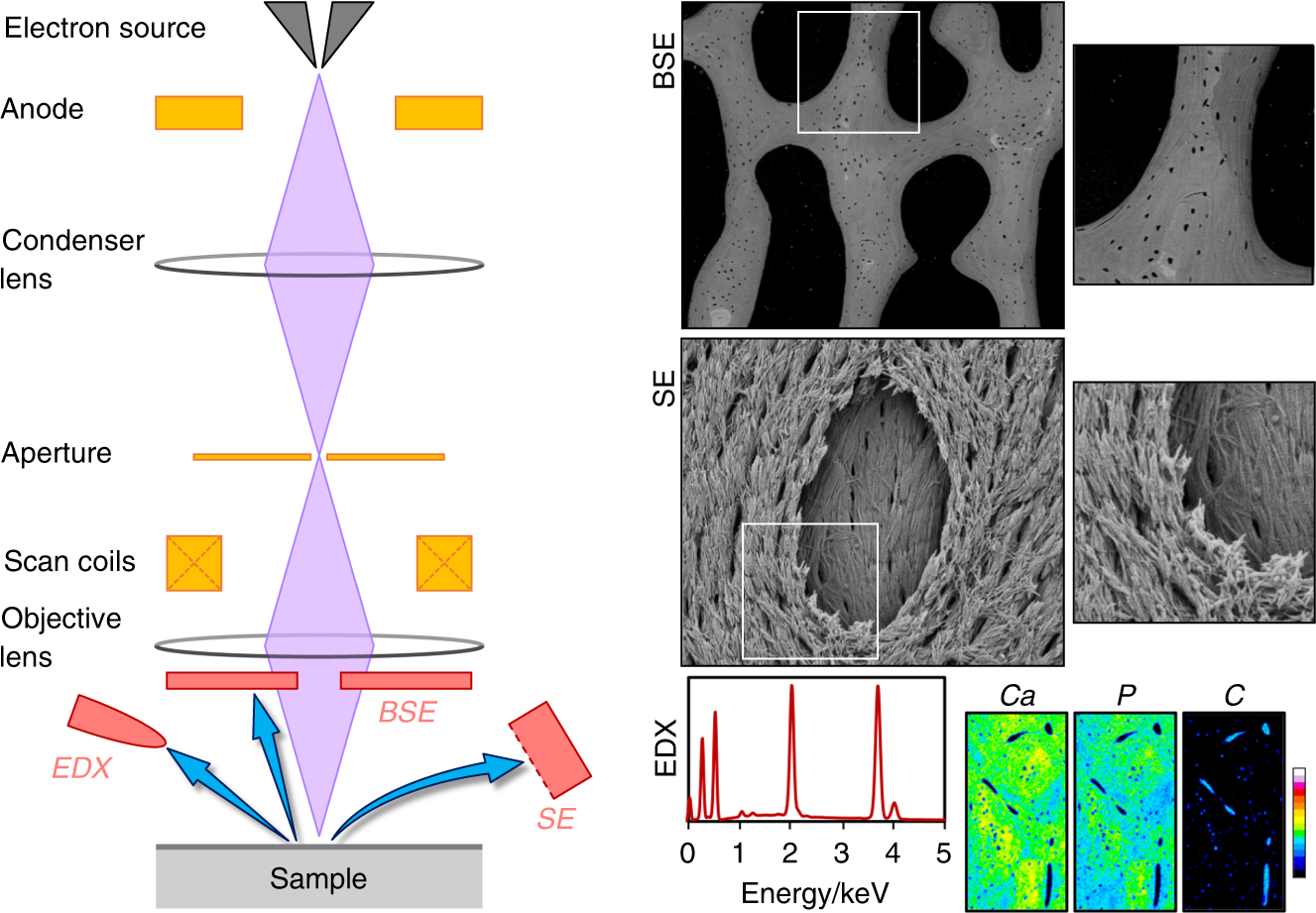


Fig.S2 Schematic diagram of the structure of SEM and the typical results ^[5,6]^

Fig. S3 SEM images and the coordinate values of the labeled points (A, B, C, D, ARR1~4) and target point(T) in SEM system

Fig. S4 Interface of the navigation part (a) and scan part (b) of AFM operation software

Fig. S5 Interface of the homebrew software explored for the positioning system: a given set of coordinate values of labeled points and target point in SEM (ⅰ) and AFM (ⅱ) system were displayed.

**Reference**

1. Y. Chu, S. Jing, Y. Zhao, “Morphological control and kinetics in three dimensions for hierarchical nanostructures growth by screw dislocations,” Acta Materialla, vol.162, pp.284-291, 2019.
2. S. Meng, F. Huang, “Synthesis, crystal structure, and photoelectric properties of a new layered bismuth oxysulfide,” Inorganic Chemistry, vol. 54, no. 12, pp. 5768-6773, 2015.
3. P. Eaton, P. West, Atomic Force Microscopy, Oxford University Press:2010.
4. T. Sun, Operation Manual of Bruker Icon Scanning Probe Microscopy, 2nd Edition, 2019.
5. F. Shah,, K. Ruscsák, “50 years of scanning electron microscopy of bone—a comprehensive overview of the important discoveries made and insights gained into bone material properties in health, disease, and taphonomy,” Bone Research,vol.15, pp.7-25,2019.
6. G. Joseph, N. Dale, M. Joseph, R. Nicholas, J. Scott, D. Joy, Scanning Electron Microscopy and X-Ray Microanalysis (4th Edition), Springer, 2018.
